# Integration of robotics as standard surgical procedure during residency training for visceral surgery using a balanced scorecard (BSC)

**DOI:** 10.1007/s11701-025-02833-0

**Published:** 2025-10-02

**Authors:** Jessica Stockheim, Susanne Boehm, Mihailo Andric, Aristotelis Perrakis, Roland S. Croner

**Affiliations:** 1 Department of General, Visceral, Vascular, and Transplant Surgery, Otto-Von-Guericke University Magdeburg, Leipziger Str. 44, 39120 Magdeburg, Germany; 2 Medical Process Management, Medical Faculty, Friedrich-Alexander-University Erlangen-Nürnberg, Erlangen, Germany

**Keywords:** Robotic surgery, Training, Management, Standard surgical procedure

## Abstract

Advancements in robotic assistance systems increased the number of robotic procedures. Successfully integrating robotics requires structured, effective management strategies and comprehensive training programs. The study aimed to develop a Balanced Scorecard (BSC) for the "Robotic Curriculum for young Surgeons" (RoCS) to establish robotic surgery as a standard surgical procedure during residency training for visceral surgery’. Strategic goals, derived from the department’s vision, mission, and strategy, were assigned to four perspectives: (1) financial, (2) patient, (3) internal processes, (4) learning and development. Key performance indicators (KPIs) and their allocation to strategic goals were assessed through expert interviews and visualized in a strategy map. Data analysis was performed retrospectively comparing 2023 to 2022. Twenty KPIs were validated as suitable for tracking progress toward the 17 established strategic goals. Four performance indicators were considered susceptible to subjective influences. Expert interviews identified two additional KPI recommendations. The BSC and strategy map show the KPI relationship and their impact on strategic goals. Yearly comparison showed an overall improvement of eight KPIs. The RoCS-BSC represents an adaptable management tool tailored to fulfill the healthcare system's requirements for implementing robotic-assisted surgery, covering patient care, surgical quality, education, and economic considerations. This study places clinical training in visceral surgery within an economic context by integrating healthcare system components. The RoCS-BSC offers a supporting framework to facilitate the systematic integration of robotic operations as a standard procedure in the surgical training of future generations.

## Introduction

The use of robotic surgical assistant systems in visceral surgery has grown rapidly, with competing systems emerging, thereby increasing both the importance and frequency of robotic procedures [1, 2]. Clinical training primarily involves procedure participation and career paths to achieve expert proficiency, evaluated through patient outcomes and technical performance metrics. Currently, there is no unified approach for incorporating robotics into surgical training or how to address educational requirements [3]. In 2020, the Department of General, Visceral, Vascular, and Transplant Surgery, University Hospital (UH) Magdeburg implemented the ‘Robotic Curriculum for young Surgeons’ (RoCS) to attain foundational proficiency in robotics during residency [4]. RoCS was integrated into clinical routine using a multimodal structure guaranteeing patient safety [4, 5]. Skill acquisition is assessed using the Ottawa Surgical Competency Operating Room Evaluation (O-SCORE), documented by trainers after each robotic procedure involving a novice participant, evaluating the capability to independently perform designated tasks [4, 6, 7]. The NASA Task Load Index (TLX) assesses subjective workload experienced during a given task [8, 9]. Comprehensive feedback is exchanged between trainer and trainee postoperatively (“bidirectional feedback”) [4, 6]. Evaluation includes trainee self-assessment and teaching satisfaction. RoCS is adaptable to facilities’ resources, making it unique as robotic systems gain significance [4].

The rapid adoption of robotic surgical systems has outpaced management tool development, particularly in training programs. Many hospitals lack structured frameworks to assess clinical usage and resident education, presenting an opportunity to apply the Balanced Scorecard (BSC) methodology, following Kaplan and Norton's approach [10–12]. It is employed for strategic management, and performance measurement in various fields and has proven effective for healthcare [11–13]. Four standard perspectives (finance, customer, internal processes, learning and growth) surround the centered vision, mission, and strategy of an organization and link them to the derived strategic goals and associated key performance indicators (KPIs). The evaluation of the four operational domains offers a comprehensive perspective on the overall efficiency of a business (or surgical division). Strategies are coordinated plans that guide organizational decision-making and establish a departmentally harmonized approach [13, 14]. The BSC enables organizations to be guided by strategic key performance indicators (KPIs), moving beyond financial metrics, offering a comprehensive perspective of efficiency, and identifying areas for improvement [15]. The BSC provides a balanced view of the organizational performance, while the strategy map visualizes KPI interconnections. The strategy map functions as a transparent medium for communication for employees [11, 13, 16]. In the healthcare context, particularly in hospital and surgical settings, the BSC perspectives are frequently adapted to reflect sector-specific requirements. Previous publications have presented different BSC applications. Onetti applied it to day surgery as a business decision-making tool for safety, cost-effectiveness, patient satisfaction, and surgical quality [17]. Amer et al. used the BSC to identify affected healthcare areas during the COVID-19 pandemic [18]. Khadge et al. used a BSC to improve postoperative recovery through anesthesiologists’ feedback [19]. Abdullah et al. evaluated a digestive endoscopy center, resulting in increased revenues, patient satisfaction, facility use, and training targets [20]. Fugaça et al. implemented the BSC in nursing, identifying 32 indicators [21]. Koumpouros analyzed the Greek public health system, revealing system gaps and improvement opportunities [22]. Curtright et al. demonstrated that Mayo Clinic’s complexity required strategic performance management [23]. They linked Mayo Clinic’s vision, primary values, core principles, and day-to-day operations through a BSC, monitoring KPIs on a regular basis and sharing data via intranet [23]. Furthermore, the BSC has succeeded in correlating human resource strategies with healthcare performance [13, 24].

The BSC has not yet been specifically adapted for robotic surgery training. It could provide the framework needed to establish robotics [11, 13]. This study sought to design a BSC to effectively integrate robotics as a standard surgical procedure during residency in visceral surgery, referencing RoCS. The primary objective was to develop a viable BSC for surgical departments that supports strategic planning, goal-oriented governance, and performance assessment. Consequently, this study aimed to merge clinical training in robotic surgery with strategic, operational, and economic dimensions of healthcare, illustrated in a strategy map.

## Material and methods

The methodological steps undertaken to develop the RoCS-specific BSC and evaluate its operational functionality are illustrated as a synopsis in Table 1.

**Table 1:**
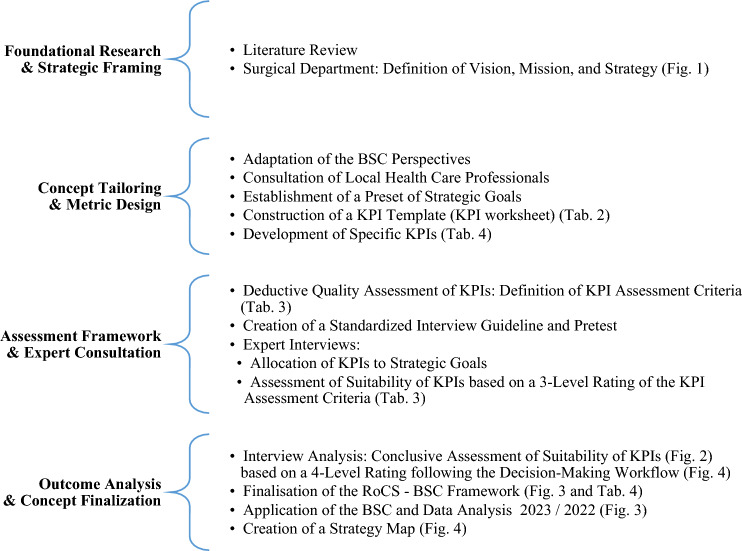
Methodological overview of the RoCS-BSC development

### BSC framework and vision

**Fig. 1:**
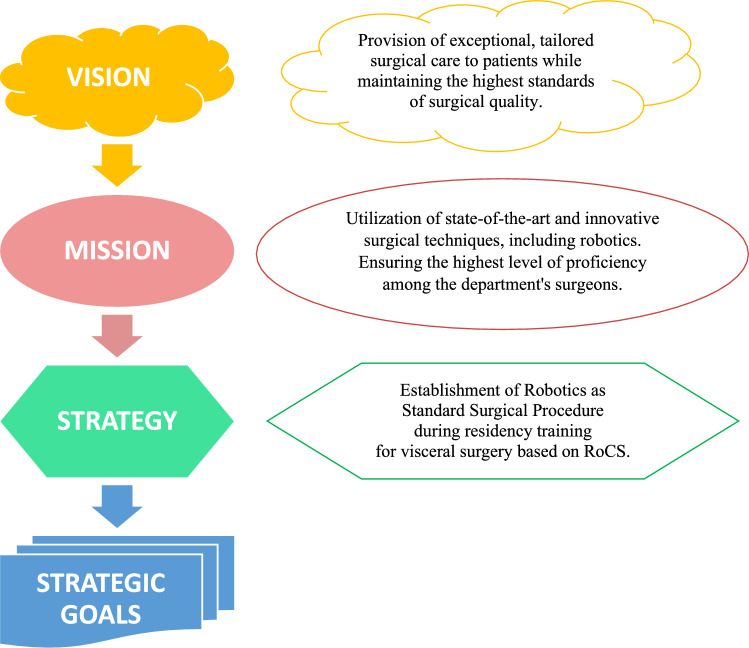
Overview of the Balanced Scorecard's hierarchy of Vision, Mission, Strategy, and Strategic Goals as applied to RoCS

The BSC framework was constructed through a comprehensive review of existing literature and a structured process that integrated theoretical BSC principles with a step-by-step methodology, utilizing, adapting, and incorporating recommended, standard performance indicators [16, 25–27]. Strategic goals—comprising critical and decision-making objectives—were defined in alignment with the vision, mission, and strategy of UH Magdeburg (Fig. 1) [27]. The department’s vision is to provide individualized surgical care maintaining the highest medical quality standards. This is accomplished through the mission of implementation cutting-edge surgical techniques, such as robotics, and ensuring the highest level of competency among the department's surgeons, which is attained by enhancing training structures (RoCS). Therefore, to realize the mission, the department’s strategy is the ‘Establishment of robotics as standard surgical procedure during residency training for visceral surgical’ (Fig. 1).

### Strategic goals, perspectives of the BSC and development of key performance indicators (KPIs)

Considering the four BSC perspectives and given the nature of healthcare and surgery, prioritizing evaluation of patient perspectives—rather than focusing solely on surgical service volume and profitability—supports a patient-centered approach. Furthermore, teaching and acquisition of fundamental robotic skills are essential to achieving the targeted vision and mission. Accordingly, the standard BSC perspectives were adapted to reflect these aspects: (1) financial perspective, (2) patient (instead of customer) perspective, (3) internal processes, and (4) learning and development. Three healthcare professionals established a preset of strategic goals based on the existing literature [16, 26, 27]. These goals were subsequently assigned to the BSC perspectives through structured interviews conducted by a research group.

Performance indicators (PI) were developed through consultation with the medical controlling department and the research group, resulting in a key performance indicator template (KPI worksheet) [11, 16, 26, 27]. Each KPI is characteristically defined by its parameter (e.g. “Rate of participation in the operation room (OR)”), target value (e.g. 100%), definition (“Participation in surgery is defined as the involvement of a resident in a robotic-assisted operation.”), formula (Rate of participation in the OR [%] = (Number of operations in which a resident was involved / Number of robotic-assisted visceral surgeries) * 100), and its availability (“internally”). Data collection was conducted in cooperation with the medical controlling department and, as part of the RoCS project, were captured electronically collected via RedCap®.

### Expert interviews, suitability of key performance indicators and allocation of KPIs to strategic goals

The study involved 15 German professionals with a minimum of five years of robotic surgery expertise and leadership experience. A structured interview guideline was developed to determine the suitability of each KPI. The criteria for deductive quality assessment of KPIs were derived from a literature review [16, 25, 28, 29]. Two commonly used KPI assessment questions were excluded due to irrelevance and failure to meet the SMART criteria [14]. The following six criteria were included: (1) appropriateness, (2) validity, (3) clarity, (4) functionality, (5) reliability, and (6) cost–benefit ratio. Appropriateness aims to address the key question ‘Is the metric fir for purpose?’. The specific guiding questions are: Does the metric support the implementation of the strategy of establishing robot-assisted surgery in surgical specialist training? Can the metric be clearly linked to one of the strategic objectives? [16, 25, 26, 29]. The concept of validity and significance seeks to answer the question ‘Is the metric meaningful?’. It examines in particular if it is possible to determine the achievability of the desired goal based on the measurement parameter [16, 26, 29]. Clarity addresses the question ‘Is the metric understandable?’. More specifically, it considers whether the metric is unambiguous and whether it can be interpreted clearly and consistently by different stakeholders [16, 25, 26, 29]. Functionality explores the question ‘Is the metric actionable?’. It focuses on answering the question ‘Can the metric influence employee behavior and organizational change?’ [16, 26, 29]. Reliability aims to determine if the metric is dependable by particularly assessing whether the metric can be manipulated or falsified by those responsible and whether the data collection method is sufficiently protected against falsification or bias [16, 25, 26, 29]. The Cost–Benefit-Ratio addresses the cost-effectiveness of the metric by examining whether the metric can be captured easily (in terms of collection, processing, and provision) and whether the costs of data collection, if not yet in use, are justified by the anticipated benefit [16, 25, 26, 28, 29].

The final KPI worksheet included all predefined KPIs along with their corresponding assessment criteria and served as the basis for expert evaluation. A pretest with two healthcare professionals required no interview guideline, and one-hour duration per interview was considered adequate.

Subsequently, online and in-person interviews were conducted. The voluntary and confidential nature of the interviews was emphasized. The expert interviews were digitally recorded, and the audio files were transcribed using MAXQDA ™ software. During interviews, each criterion was rated using a 3-level scale: “fully met”, “partially met” and “not met”. Additional performance indicators (PI) were collected afterwards. In the subsequent interview phase, each PI was allocated to one strategic goal, with each allocation counting as one. In case of multiple assignments, the strategic goal with the highest allocation rate (count) was considered to be the definitive strategic goal for this performance indicator. Upon completion of the interviews, data were anonymized.

**Fig. 2:**
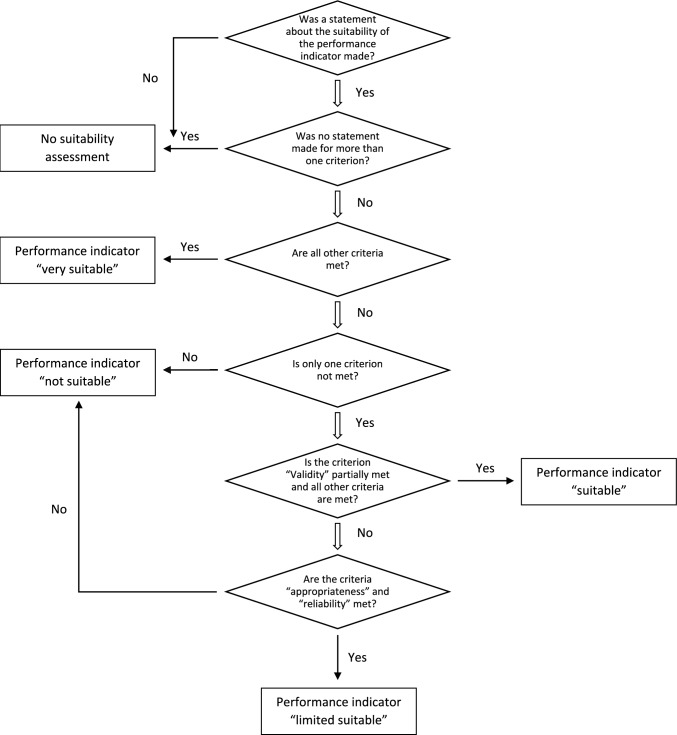
4-Level-Rating of performance indicators’ suitability (based on six criteria: 1. appropriateness, 2. validity, 3. clarity, 4. functionality, 5. reliability, and 6. cost–benefit ratio) following the workflow of the decision-making process following expert interviews

Figure 2 illustrates the KPI suitability assessment process and decision-making workflow after the expert interviews. The final suitability of each KPI was evaluated using a 4-level rating scale: “very suitable”, “suitable”, “limited suitable”, “not suitable”. If a performance indicator was not assessed at all, it was abandoned and reported as “no suitability test”. In case of inconclusive results, the decision was made by the department’s research team. The average suitability score was calculated unweighted, considering only assessments rated as “very suitable”.

### Application of the BSC, data analysis and strategy map

The years 2022 and 2023 were selected for retrospective analyses due to stabilized post-COVID circumstances. The UH’s data protection rules prohibited publishing two KPIs. The remaining KPIs were included based on the RoCS study, with a positive approval of the ethics committee. Statistical analysis was performed descriptively with percentage showing demonstrating performance status and trends. Color coding visualizes whether KPI targets were achieved (green) or not (orange). The cost–benefit ratio was not weighted. The Strategy Map concisely highlighted the cause-and-effect relationships between BSC strategic goals and KPIs.

## Results

In total, ten expert interviews (67%) were successfully performed (five in-person, five online), including four specialists, three consultants, and three heads of a general/visceral surgery department in Germany.

### Strategic goals, KPIs and their suitability

In total, 18 strategic goals were preset with 22 eligible performance indicators. Table 2 illustrates the final 17 strategic goals, 20 KPIs, and results of the comprehensive suitability assessment of KPIs. The suitability assessment showed an overall average score of 6.7 / 10 points for the 20 KPIs.

**Table 2:** Illustration of the final strategic goals with the corresponding final KPIs and results of the suitability assessment (4-Level-Rating); Abbr., Abbreviation; ^1^LOS, Length on in-hospital stay; ^2^OR, operation room; ^3^O-Score, Ottawa Surgical Competency Operating Room Evaluation; ^4^NASA TLX, National Aeronautics and Space Administration (NASA) Task Load Index

Perspective	Abbr	Strategic Goal	KPI	Suitability				
				“very suitable”	“suitable”	“limited suitable”	“not suitable”	not assessed
Financial	F1	Reduction of costs per patient case	Costs per patient case	6	1		2	1
	F2	Reduction of costs for trainees	Personnel turnover rate	7			3	
	F3	Increase of budget efficiency	Cost per Case Mix Index Count	8			1	1
	F4	Minimization of robotic training costs for trainees	Costs for robotic training	5			4	1
	F5	Optimization of cost efficiency through low idle time	Utilization rate of robotic surgical system	8			2	
Patient	P1	Guarantee of patient safety	a) In-hospital mortality b) Surgical morbidity	9				1
			c) Robotic Team-time-out rate	9			1	
	P2	Reduction of LOS^1^	LOS	7		1	2	
	P3	Increase of patient satisfaction	Rate of satisfaction	5			4	1
	P4	Improvement of postoperative performance status	Pain scale count	6			4	
Internal Processes	I1	Increase of OR^2^ process efficiency	Duration for trocar placement and docking	7		1	2	
			Stable average operation time	7			3	
	I2	Enhancement of willingness for perioperative documentation	Documentation rate of RoCS	7			1	2
	I3	Minimization of intraoperative complications	Rate of intraoperative complications	5			5	
	I4	Proven Indication for robotic approach	Rate of robotic indication	7			2	1
	I5	Clarification of communication culture	Bi-directional feedback	7			2	1
Leaning and Development	L1	Continuity of satisfactory learning effect	Satisfaction rate of trainees	6		1	3	1
	L2	Increase of practical surgical training of trainees	a) Rate of participation in the OR	7		1	2	
			b) O-Score^3^	7		1	1	1
	L3	Creation of an appropriate working and performance atmosphere	NASA TLX^4^	4		1	4	1

The PI “fluctuation rate of trainees” was allocated to the goals of the perspective "Learning and Development" twice. The “robotic training costs” and “participation in the OR” were allocated to two strategic goals, one being “reduction of training time”. Consequently, the research group determined that “reduction of training time” should be removed as a strategic goal in the BSC, as there is synergy between enhancing practical surgical training and minimizing training times. “Robotic training costs” were referred to “reduction of robotic training costs for staff”, and “participation in the OR” was assigned to “improvement of practical training” by the research group.

During expert follow-up questioning regarding additional indicators, two potential PIs were named: "bed occupancy rate in Intensive Care Units (ICU)" and "Proficiency Based Progression (PBP)".

### Critical performance indicators

The following indicators were included for framing the BSC, but were rated suitable by less than 50% of the interviewed experts:

- Training costs

The training costs for residents were rated inadequate three times in terms of feasibility and once in terms of credibility and comprehensibility. Reliability was not met once and was only partially met once. The cost–benefit ratio was not met once.

- Patient satisfaction rate

The patient satisfaction rate was only partially credible once and the criterion for reliability was not met four times. It was rated as non-functional once.

- Intraoperative complication rate

The intraoperative complication rate scored partially credible in terms of comprehensibility once, with only partial fulfillment of the criteria for functionality and reliability. Functionality was “only partially achieved” once and “not at all achieved” once. Reliability was rated as “only partially fulfilled” by four experts twice and “not fulfilled” twice. The intraoperative complication rate was criticized for being highly subjective, as complications are often subjectively documented and their definitions vary from surgeon to surgeon.

- NASA Task Load Index

The NASA-TLX was rated as “only partially credible” twice and did not meet the criterion for functionality once. Reliability was not met three times and “only partially met” once. The cost–benefit ratio was rated as “only partially fulfilled” once. It was neither credible nor reliable because of subjectivity.

### BSC and strategy map

**Fig. 3:**
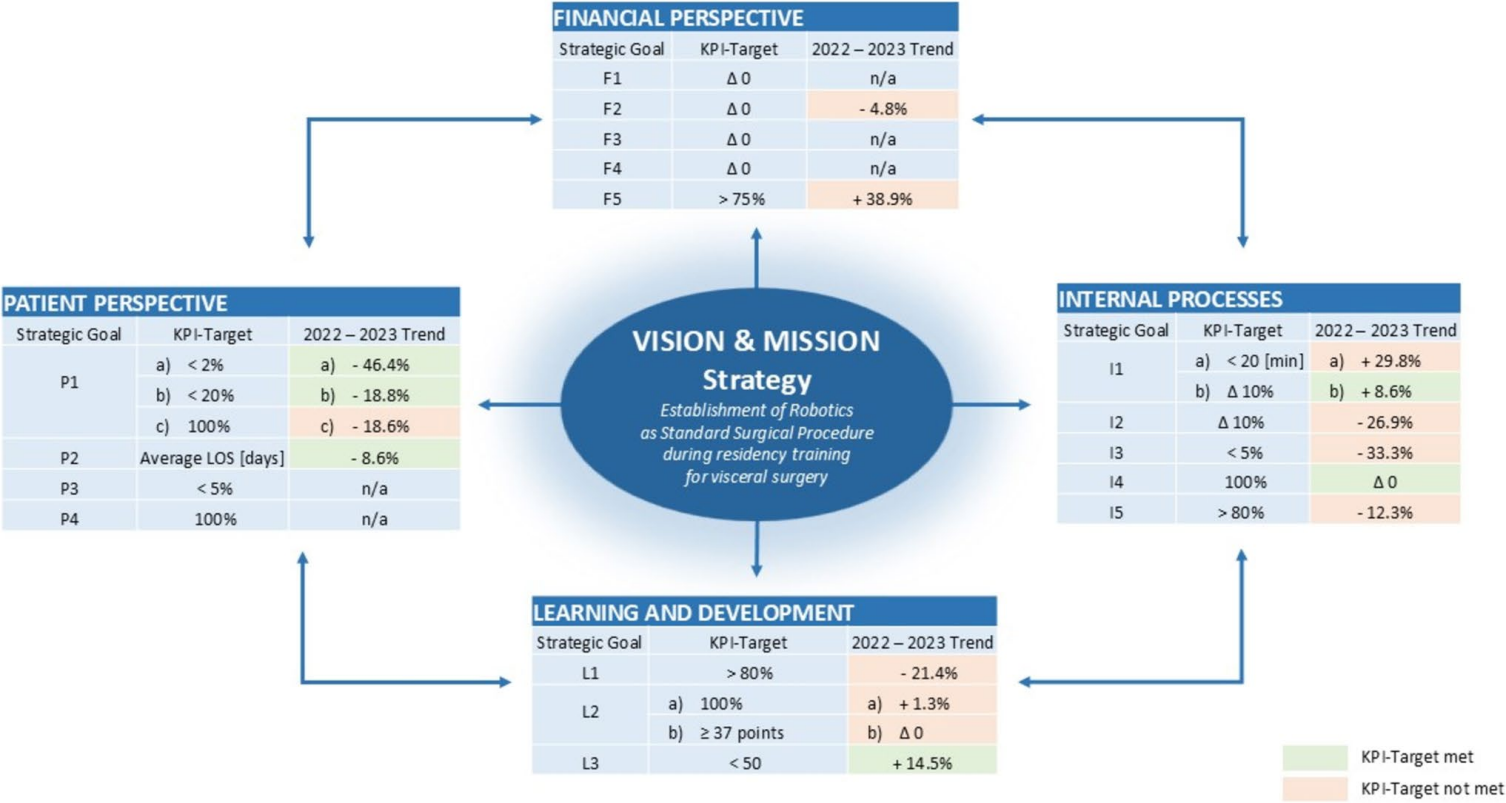
‘Robotic Curriculum of young Surgeons’ – Balance Scorecard (RoCS – BSC) with the four perspectives, their Key Performance Indicators (KPI) and trends (comparison of data for 2022 and 2023)

Figure 3 shows the final BSC with yearly data comparative analysis for 2022 and 2023. The comparison revealed a notable increase in performance from 2022 to 2023 from the financial perspective (F2, F5), internal processes (I1b, I3), learning and development (L2a), and patient perspectives (P1a, P1b, P2). In total, all 90 operations from 2022 and 125 operations from 2023 were included in the analysis, all of which were related to operations on the upper gastrointestinal, liver, pancreas and colorectal areas. Costs per patient case (**F1**) and costs per Case Mix Index Count (**F3**) were not applicable. Training costs (**F4**) were not explicitly extractable from case costs. Personnel turnover rate (**F2**) decreased from −9.1% in 2022 to −4.3% in 2023, a 4.8% decline. Robotic system utilization (**F5**) increased by 38.9% from 2022 to 2023, but did not reach 75% of the targeted procedures performed robotically.

Mean trocar placement time (**I1a**) increased from 15.8 ± 1.9 min. in 2022 to 20.5 ± 3.1 min. in 2023, a 29.8% longer duration. Mean duration of robotic operations (**I1b**) increased from 213.3 min. ± 35.0 in 2022 to 230.7 min. ± 25.6 in 2023, an 8.2% growth. Conversely, RoCS documentation **(I2**) declined from 82.5% in 2022 to 60.3% in 2023, a 26.9% reduction. Intraoperative complications (**I3**) declined by 33.3% from 14.4% in 2022 to 9.6% in 2023. The indication for the robotic approach (**I4**) remained consistent at 100% for both 2022 and 2023. Bidirectional feedback (**I5**) decreased by 12.3% from 81.3% in 2022 to 71.3% in 2023.

Satisfaction among participating trainees (**L1**) declined from 95.6% in 2022 to 75.3% in 2023, a 21.4% decline. Residents participating in the OR (**L2a**) slightly increased by 1.3%, from 62.2% to 63%. The O-Score (**L2b**) remained stable, with 26% of participants reaching a minimum of 37 points out of 40 in both years. The NASA TLX (**L3**) scored an average rise of 2.5 points, from 17.3 ± 3.3 in 2022 to 19.8 ± 3.1 in 2023, a 14.5% increase.

In-hospital mortality rates (**P1a**: 2.8% in 2022, 1.5% in 2023, a 46.4% decline) and surgical morbidity rates (**P1b**: 8% in 2022, 6.5% in 2023, a 18.8% decrease) remained low. Robotic team time-out (rTTO; **P1c**) decreased from 86% in 2022 to 70% in 2023, a 18.6% decline. Average length of stay (LOS; **P2**) decreased from 14 days ± 1.8 out of 14.8 planned days of LOS in 2022 to 12.8 days ± 2.5 out of 13 planned days in 2023, an overall 8.6% decrease. Patient satisfaction (**P3**) and pain scale (**P4**) were not applicable for 2022 and 2023, respectively.

**Fig. 4:**
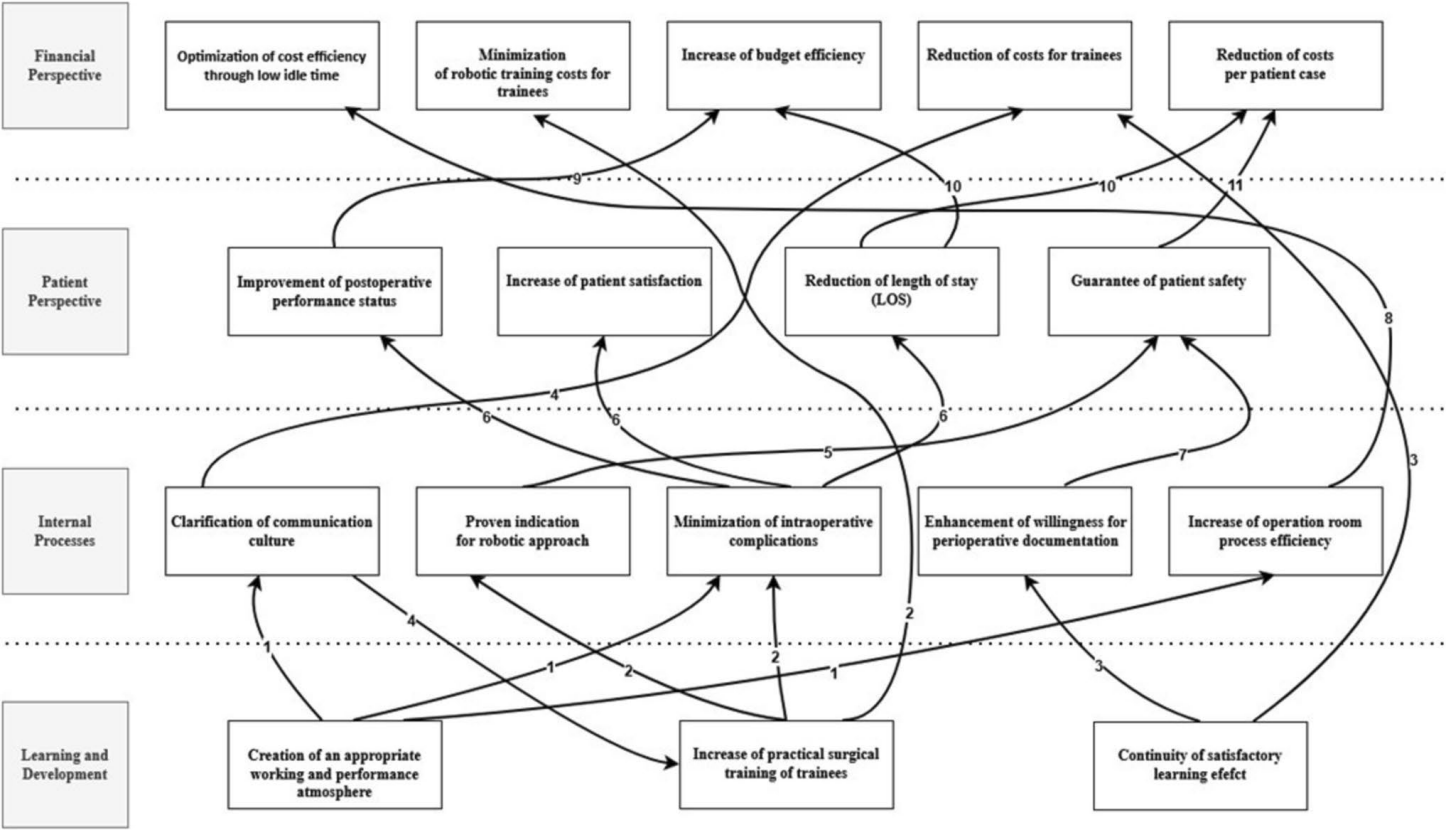
Strategy Map related to the Balanced Scorecard of ‘Robotic Curriculum of young Surgeons’ (RoCS); The number assigned to the lines connecting strategic goals refers to the number of explanations: 1) Creating and ensuring an appropriate working and performance atmosphere helps to minimize intraoperative complications. At the same time, this increases surgical process efficiency and clarifies the communication culture. 2) An increase in practical surgical training leads to a reduction in intraoperative complications and contributes to the safety of a proven indication rate. At the same time, it helps to minimize personnel training costs. 3) A continuous satisfactory learning effect contributes to an increase in perioperative documentation willingness and reduces personnel costs as measured by the fluctuation rate. 4) A more precise communication culture promotes the positive well-being of trainees and should reduce the fluctuation rate and the associated personnel costs. 5) The proven indication rate guarantees patient safety. 6) Minimizing intraoperative complications improves the postoperative performance status and increases patient satisfaction. Simultaneously, DRG-specified average LOS can be adhered to by reducing intraoperative complications. 7) Increased willingness to perform perioperative documentation helps guarantee patient safety. 8) The increased OR process efficiency leads to the optimization of cost efficiency by minimizing idle times. 9) Improvements in patients’ postoperative performance status contribute to an increase in budget efficiency. 10) A reduced LOS helps reduce case costs per patient and increases budget efficiency. 11) Ensuring patient safety helps reduce case costs per patient and increases budget efficiency by generating fewer material and operating costs that are part of the case cost

Figure 4 illustrates strategy formulation and implementation context (Strategy Map). The visualization of vision and strategy demonstrates how individual goals are transparently interdependent across perspectives.

## Discussion

The BSC application in healthcare has been diverse, ranging from day surgery optimization to healthcare system evaluation [17, 18, 20–23, 30]. Studies have demonstrated its effectiveness in improving patient care, operational efficiency, and strategic management [12, 17, 18, 20–23, 30]. It is recognized as one of the most successful strategic management tools with the potential to change clinical practice [19, 21, 30]. This study introduces the first BSC and strategy map designed to establish robotics as a standard surgical procedure for residents. The BSC principle is also used for evaluating professional competencies and talent management, making it particularly relevant for training the next generation of surgeons in an increasingly robotic-driven field [30]. Currently, robotic surgery lacks strategic management concepts for basic training, but it is essential that robotic novices participate regularly in daily robotic [2, 4, 31]. However, the context of robotic surgery is complex considering diverse stakeholders. In light of the aforementioned, our study identified 20 KPIs aligned with 17 strategic goals across financials, patients, internal processes, and learning domains. The 2023/2022 comparative BSC analysis revealed improvements in financial metrics (reduced turnover, increased robotic system use), internal processes (fewer complications), learning (more resident OR participation), and patient outcomes (lower morbidity and mortality). However, challenges persisted in some areas with worsened KPI results, including longer trocar placement times, decreased documentation, and lower trainee satisfaction. Referring to the strategy map, we observed an unfortunate 21.4% decline in residents’ satisfaction and a 26.9% reduction in documentation, highlighting the interconnection of these KPIs. Conceptual staff rotation may have contributed to these results, as it often involves younger, less experienced residents with varying technical skills and prior experience, reflecting ongoing learning curves that can influence procedural efficiency, trainee satisfaction and motivation for documentation, potentially affecting future annual comparisons.. Patient safety remained assured regarding morbidity and mortality, but the rate of the specific educational team time out (rTTO) decreased, further reflecting KPI interconnection. Lower trainee turnover may help reduce recruitment costs, although additional evidence is required. As outlined in point 3 of the strategy map, personnel expenses could be mitigated by retaining trainees through consistently positive earning experiences, thereby reducing the staff fluctuation rate. Consequently, based on this BSC analysis, addressing the underlying causes of reduced satisfaction is crucial to improve interacting KPIs. Furthermore, the KPI “rate of robotic indication” may appear to be of negligible consequence at first, but documenting clear indications for robotic surgical approaches is vital for quality assurance and medicolegal liability, especially in the event of complications [32–35]. Establishing this as a KPI promotes a quality and safety culture environment [32–35]. Proper documentation of robotic indications reflects compliance with clinical guidelines and ensures state-of-the-art medical practices using technology based on clinical necessity rather than solely economic factors.

Operational recommendations for the implementation of a BSC in other robotics centers include the clear assignment of responsibilities for data collection and analysis, close collaboration with the medical controlling department, and the establishment of regular data reviews and evaluations (e.g., monthly or quarterly). The BSC should be updated periodically, e.g. every two or five years, based on the collected data to better understand its dynamics and to continuously refine the strategic approach. Although there is no universal blueprint -particularly given the international variability of healthcare systems, for example, regarding the financial perspective – selected variables and KPIs should, where possible, be standardized internationally (e.g., morbidity, mortality, LOS) to allow benchmarking and cross-center evaluation. Critical Key Performance Indicators and Suggestions for KPIs.

The ethical principle of patient focus during medical activities is highly relevant and cannot be fully addressed through objective parameters (e.g., operation time etc.) or surgical outcome parameters, though these are essential for tracking patient safety [36]. The KPIs patient satisfaction (P3) and postoperative performance status (P4) represent the individual patient perspective and, based on the authors’ opinion, should be retained.

The KPI “intraoperative complications” (I3) was criticized for subjectivity. While the Clavien-Dindo classification is widely accepted for postoperative complications, standardized reporting of intraoperative complications remains considerably debated [37, 38]. Sayegh et al. revealed poor adoption of existing intraoperative adverse events (iAE) grading systems [38]. Our strategy map displays the relationship between intraoperative complications and financial level, given that iAEs are associated with increased hospital costs reported by Garbens et al. [37]. However, this KPI’s needs a precise definition, appropriate documentation, and surgical consensus. In the authors’ opinion, as it is a crucial quality parameter for training and patient safety, it should remain within the BSC framework.

In light of training robotics, the interaction between humans, technology, and working atmosphere impacts patient safety [8, 9]. The NASA TLX represents an important factor for learning and development. Factors negatively affecting workload, posing a risk to patients through an increased rate or severity of errors, can be investigated from a personnel perspective, particularly surgeons themselves [8, 9]. During challenging tasks that require high effort or are physically demanding, a positive workplace culture with effective communication can help reduce perceived e.g. mental and temporal demands, improve performance and lower frustration levels, thereby maintaining safe overall workload levels. Therefore, the authors recommend continued use of it as a meaningful KPI.

Officially, training costs are included in diagnosis-related groups (DRGs) [39]. However, in practice, it would be erroneous to assume that they are necessarily covered in this manner. The KPI “robotic training costs” (F4) is therefore considered a relevant functional indicator, with the RoCS concept being a cost-effective and sustainable training approach when integrated into regular working hours.

To address KPI criticality, standardized adverse event definitions, validated satisfaction instruments, and NASA TLX administration training will be incorporated in future internal updates and BSC revision.

ICU occupancy rate was suggested to be an important indicator due to its financial impact. Minimally invasive procedures, including robotics, are generally associated with lower morbidity and a shorter hospitalization, lowering ICU demand [1, 5, 40]. Surgical training should not affect ICU capacity, which impacts patient health, as measured by KPI P1, P2, I1, and I3. Our strategy map shows these relationships, and this indicator could be discussed in future BSC updates. Furthermore, PBP was suggested as a potential indicator for learning [31, 41, 42]. RoCS considers procedural progress in basic robotic skills, including simulation training prior to operating the console and focusing on the performance of procedural steps rather than complete procedures [4]. Therefore, this indicator was not considered helpful as it is an essential element of RoCS.

### Limitations

This study is limited by its retrospective design to evaluate the feasibility and applicability of the RoCS-BSC through internal KPI data analysis over a two-year period with a restricted sample size and single institutional setting. Certain KPIs could not be disclosed due to legal restrictions, limiting publication scope. Additionally, incomplete interview participation of experts could have introduced bias, thereby compromising the data robustness.. Nevertheless, the findings support internal management, process optimization. As the RoCS-BSC and strategy map reflect the department-specific vision, results may vary across institutions. While analyzing specific procedures could enhance optimization, this study focused on a comprehensive robotic program across all procedures. Furthermore, various management frameworks exist in healthcare; for example, Lean Six Sigma applies the DMAIC circle: Define, Measure, Analyze, Improve, and Control [43]. Although BSC is widely used, its specific impact on surgical training, remains inconclusive [13]. Additional variables, including case mix, staff rotation, trainee seniority, and changes in equipment, may have significantly influenced the observed differences between the two years and represent potential confounders. Nonetheless, the implementation of robotics and robotic training is and remains a complex, multifaceted process, making it challenging to provide a comprehensive overview and to develop an effective management tool.

## Conclusion & Perspectives

Together, the BSC and Strategy Map offer a structured management approach that aligns the needs of key stakeholders – residents, faculty, hospital administration, and patients. This study contextualizes next-generation robotic visceral surgery training within RoCS by integrating essential healthcare components in a targeted, resource-conscious way. The RoCS-BSC provides a flexible, supporting framework to balance patient care, surgical quality, education, and economic requirements in a modern academic surgical department. Teaching hospitals, in particular, may benefit from improved training outcomes while maintaining high standards in surgical education and patient care. Future efforts could expand the management toolkit, emphasizing visualization, strategic alignment, and the application of artificial intelligence, and machine learning.

## Data Availability

The data supporting the findings of this study are available from the corresponding author upon request. Some data are not publicly available due to medicolegal restrictions of the University Hospital Magdeburg.

## Abbreviations

BSC: Balanced Scorecard
KPI: Key Performance Indicator
NASA TLX: National Aeronautics and Space Administration Task Load Index
RoCS: Robotic Curriculum for young Surgeons
